# The RELIANT checklist: a novel approach to revealing implicit factors in HTA deliberations

**DOI:** 10.1016/j.hpopen.2025.100149

**Published:** 2025-10-12

**Authors:** Clara Monleón, Hans-Martin Späth, Carlos Crespo, Mondher Toumi

**Affiliations:** a Health Systemic Process, EA 4129 Research Unit, University Claude Bernard Lyon 1, University of Lyon, Lyon, France; b Statistical Department, University of Barcelona, Avinguda Diagonal, 643, 08028 Barcelona, Spain; c Aix Marseille University, Public Health Department, EA3279 Marseille, France

## Abstract

• Implicit factors may play a role in the HTA deliberative process.
• Current guidelines do not account for implicit factors in a systematic way.
• To assist HTA practitioners in their appraisals, we developed the RELIANT checklist.
• This is the first checklist designed to help HTA practitioners reflect on implicit factors that may influence the HTA deliberative process.

## 1 Introduction

A deliberative process can be defined as an exchange of information between a group of actors, to critically review an issue, build consensus and subsequently inform decision making [1].

Deliberation in Health Technology Assessment (HTA) is the informed and critical examination of an issue and the weighing of arguments and evidence to guide a subsequent decision [2]. It is considered an effective approach for integrating complex evidence, diverse values, and multiple perspectives [3].

There are some common factors that have been the focus of the HTA. Those usually are safety, efficacy, and cost-effectiveness of the intervention [4,5]. However, even among similar European healthcare systems evaluating the same health technologies, different HTA recommendations have been issued. This suggests that the HTA deliberative process may not rely on evidence but can also be influenced by other key considerations in decision-making such as political, social, or ethical elements [[6], [7], [8], [9]] which are not always evidence-based [10] and are not always explicitly recognized. Some of these factors remain unofficial, often due to their unconscious nature, which can affect both the transparency and legitimacy of decision-making [6,7]. These factors have been referred by the authors of this article as “implicit factors”, defined as all non-defined elements that are not explicitly collected or described in the HTA guidelines and that may influence the HTA deliberative processes and the subsequent HTA recommendations [11,13].

There are several initiatives and frameworks aimed at promoting transparency, inclusivity, and impartiality in HTA deliberations. These initiatives seek to standardize and structure deliberative processes to ensure that all relevant elements are systematically considered and resolved. However, current frameworks do not sufficiently account for implicit factors [14,15]. An assessment of the influence of implicit factors in HTA deliberative processes across five European countries revealed that their impact varies depending on the national context. The lack of explicit acknowledgment or management of these factors poses a challenge to achieving legitimate and consistent decision-making across different HTA systems [11].

In response, policymakers have been encouraged to support research that enhances understanding of biases by developing tools to mitigate them and reduce healthcare disparities [16]. This context motivated the development of a practical checklist for HTA practitioners, designed primarily to identify and address implicit factors. Intended to support HTA processes for medicines, the checklist ensures that key considerations, including those often overlooked, are systematically assessed, thereby strengthening the transparency, objectivity, and legitimacy of HTA deliberations and their resulting recommendations.

## 2 Methods

**Fig. 1:**
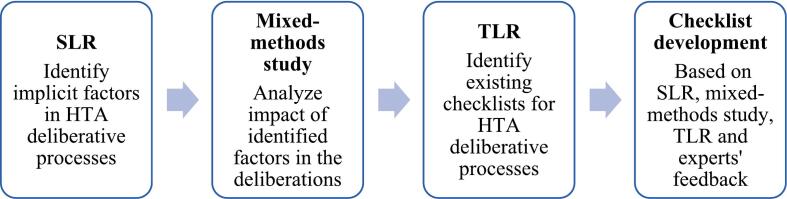
Development process of the checklist.

To develop our checklist, we followed a stepwise approach (Fig. 1). The first step was to perform a Systematic Literature Review (SLR) [12] to identify the implicit factors influencing HTA deliberative processes in five European countries (France, Germany, Italy, UK, Spain).

After identifying these factors, we confirmed and analized their impact through a mixed-methods study [11], assessing how they shaped and influenced deliberations in the five countries in scope.

Building on these findings, we carried out a targeted literature review (TLR) to identify existing checklists designed to support HTA deliberative process. Together, these research steps informed and guided the development of our checklist.

Our tool was finally assessed by eight experts who provided feedback on the different sections of the checklist. A description of the methodology of the TLR and the development of the checklist is provided in the Appendix A, Appendix A of the Supplementary Material.

## 3 Results

### 3.1 Findings from previous research

#### 3.1.1 SLR

The SLR identified a range of implicit factors, such as ethical considerations, psychology, equity, politics, society, culture, functional role, disease perception and decision-maker experience, that influence HTA deliberations across Europe. The findings revealed significant variability in how these factors are considered, often in an unstructured and non-transparent manner. This lack of consistency and visibility highlighted the need for a structured framework to make implicit factors explicit, ensure they are consistently addressed, and standardize their documentation. This, in turn, would strengthen the rationale for explicitly identifying and considering these influences in a checklist.

#### 3.1.2 Findings from the mixed-methods research

The findings from our mixed-methods research deepened the understanding of how implicit factors influence HTA deliberative processes and confirmed their significant impact. Respondents emphasized the need for more structured deliberation, clearer guidance, and practical tools to improve the transparency, methodology and the stakeholder involvement in the process. These insights underscored the importance of establishing a structured framework to systematically address implicit considerations, reinforcing the value of explicitly recognizing the elements that shape HTA decisions and thereby enhancing the overall transparency and legitimacy of the process.

#### 3.1.3 TLR – Appraisal of the HTAi/ISPOR Task Force checklist

The last step before proceeding with the development of our checklist was the TLR.

Four publications were retrieved from which one was duplicated.

Among the remaining three, a full-text screening was performed and just one article was included: the joint HTAi/ISPOR Task Force checklist published in June 2022 [2]. The reason for excluding the other two articles [15,17] was that the content did not address the research question and therefore they did not present a checklist assisting the HTA deliberative process.

An appraisal of the checklist from the joint HTAi/ISPOR Taskforce was performed by assessing the relevance of the content in accordance with the “implicit factors” (Appendix A, Appendix A) and by mapping its content against the findings from our mixed-methods research. We checked if it was alluded through different terms: "implicit"; "informal";"non-explicit factors". We also leveraged any section that could potentially address the implicit factors, to expand it further in our tool.

Our appraisal of the checklist revealed several key strengths and limitations.

A key strength is its clear definitions of deliberation and the deliberative process, providing a structured framework across six sections to support HTA deliberations. However, the achievement of its objectives is difficult to assess, as they are not easily measurable.

While the checklist seeks to make assumptions, arguments, and values explicit, it does not directly address implicit factors, which may influence recommendations and affect legitimacy, fairness, and objectivity. Additionally, it overlooks the consideration of decision-making approaches (such as consensus or voting) between supporting the process and communicating outputs.

Mapping the checklist against our mixed-methods research demonstrated its relevance and broad alignment with current best practices, while also highlighting the need to develop a more comprehensive checklist that explicitly accounts for implicit factors.

### 3.2 Development of the RELIANT checklist: a tool to address implicit factors in the HTA deliberative process

Given the absence of checklists in the literature that account for the implicit factors influencing the HTA recommendations, we developed the RELIANT **(RE**vea**L**ing **I**mplicit f**A**ctors i**N** H**T**A) checklist. RELIANT is a name that resonates with the objectives of this checklist since its implementation aims to address the potential implicit factors and biases that may compromise the credibility and trust on the HTA deliberation.

The RELIANT checklist (Table 2) is introduced as the first tool specifically designed to address these implicit factors within HTA. It serves as a practical resource to acknowledge and elucidate the implicit factors shaping the HTA deliberative process for pharmaceuticals, including the meeting dynamics surrounding this process. In HTA, deliberation occurs from horizon scanning to monitoring, assessment, appraisal, and evaluation of issued recommendations. The RELIANT checklist should be used prospectively by any HTA practitioner, whether directly or indirectly involved in deliberation, in order to raise awareness of potential implicit factors that may compromise the fairness of the final recommendation [18].

**Table 2:** Final version of the RELIANT checklist following the experts‘review

RELIANT checklist © 2025 by Clara Monleón is licensed under Creative Commons Attribution 4.0 International. To view a copy of this license, visit https://creativecommons.org/licenses/by/4.0/

The audience for this checklist is HTA experts who have a direct or indirect involvement in the HTA deliberative process.

The checklist has seven sections: Perspectives, Objective Factors, Committee Meeting Dynamics, Personal Interests, Previous Experiences, Context, and Cognitive Biases. It contains 16 questions, each of them having a different range of options of answers.

The first section, “Perspectives”, aims to gather the perspectives that have been considered in the deliberative process such as the health insurer/payer perspective, the societal perspective or other.

Section B, “Objective factors”, addresses the objective factors that HTA experts need to evaluate and calibrate according to their importance within the HTA process. This section begins to consider factors that may be either explicit or implicit, depending on the HTA body and its methodology. Specifically, it focuses on clinical burden and unmet need**.**

The following section, “Committee meeting dynamics”, aims to check on the preparedness for the meeting and clarity of the data and topics for discussion. For example, it asks if the HTA expert in question received all the necessary information to take a decision. It also aims to evaluate if there were any dominant perspectives and if the way of reaching a conclusion may affect the expert performing the checklist’s perspective. This section prompts to the reflection on the way of working.

Section D or “Personal interests” focuses exclusively on the personal interests and how this can affect the expert’s perception. As opposed to section C that covers the group meeting dynamics, this part focuses on the personal factors that can represent a bias and deserve to be analysed separately.

The next part, section E or “Previous experiences”, is about the previous experiences of the individual reviewing the checklist and their impact on their perception, for example any previous experience with the manufacturer or the disease that could affect his appraisal.

Section F, “Context”, aims to consider any kind of contextual influence that might have affected the recommendation. Any influence from society, culture, media, politics, healthcare professionals, manufacturers, and/or health administration should be captured in this section.

Finally, section G or “Cognitive biases”, dedicates special attention to the different cognitive biases that might have impacted the expert’s judgment. These biases are related to emotion, first impression, selective attention, deference to authority, belief, consistency tendency, false consensus effect, framing effect, groupthinking, novelty, optimism, overconfidence and scientific inbreeding. Definitions for each bias are provided within the checklist itself.

The checklist also provides space for additional observations not explicitly captured in the individual sections.

### 3.3 Assessment of the checklist

The profile characteristics of the participants who reviewed the original checklist (Appendix A, Appendix A) is detailed in Table 1a in the Supplementary Material.

The main comments were about making the checklist a practical tool, short, concise, and easy to deploy (Appendix A, Appendix A).

All eight experts found the checklist useful, and six of them would use the tool prior to the appraisal whereas one expert thought it could be used during the deliberation and another expert saw the value of using the tool retrospectively, meaning after the appraisal.

The arguments supporting its use prior to the deliberation were that it would allow to think prospectively on all the implicit factors that can impact the recommendation and therefore this would allow the HTA practitioner to be conscious and thoughtful about them. This in turn could be a way to mitigate their influence.

It was suggested to define the target audience expected to run this checklist as well as the type of health technology (medicines for the purpose of this research).

The experts acknowledged the need for the validation of the tool. Overall, the checklist was perceived as a valuable tool, exhaustive and practical with the potential to play a key role in future HTA processes.

Their comments and suggestions were incorporated leading to a shorter second and final version that was shared for final comments and endorsement. Their responses are detailed in Appendix A, Appendix A from the Supplementary Material.

Once we had the final version, the checklist was called RELIANT (Table 2).

## 4 Discussion

The findings of our previous mixed-methods research [11] suggest that implicit factors may play a role in the HTA deliberative process. However, current guidelines do not account for implicit factors and in consequence their impact has not been systematically mitigated [11,13].

As per the HTAi/ISPOR Task Force, “well conducted deliberative processes can make explicit the arguments that are entailed when assessing health technologies” [19]. We believe that to make explicit these arguments, implicit factors must be called out. The use of checklists by the chair and committee members of HTA bodies is encouraged as an explicit approach. Similarly, HTA should be an unbiased and transparent exercise encompassing tools to mitigate the influence of cognitive biases [19].

To assist HTA practitioners in their individual and collective assessments and appraisals, we developed the RELIANT checklist. To our knowledge, this is the first checklist to support the HTA deliberative process by guiding the HTA practitioner to reflect on the implicit factors that may influence the HTA deliberative process and recommendations. It fills the existing gap on the implicit factors that other checklists have not addressed so far [2]. While the objectives of the HTAi/ISPOR Force checklist focus on the governance and the stages of an HTA process, the RELIANT checklist focuses mainly on the implicit factors influencing deliberation.

The RELIANT checklist serves as a structured guide to assist HTA practitioners in identifying and address various implicit factors throughout the HTA process. Designed as a practical tool, it aims to raise awareness and provide clarity regarding the implicit elements that influence the deliberative process in HTA, especially in the context of pharmaceuticals.

The RELIANT checklist should be used prospectively by any HTA practitioner directly or indirectly involved in the HTA deliberation, prior to reaching a final recommendation to increase awareness of potential implicit factors that may jeopardize the transparency and legitimacy of the process. HTA committees should allow for some time to deploy this checklist and reflect on the factors identified.

At national and regional levels, HTA deliberation seems to be heterogeneous and not fully explicit. The RELIANT checklist might be a vehicle to further harmonize the appraisal step, revealing and acknowledging the role of implicit factors in addition to the explicit factors already described in the HTA guidelines.

The importance of the development of methods to address biases, and in consequence improve decision-making, has been pointed out by D. Kahneman in his book “Thinking, fast and slow” [20] in which he encourages to use checklists to control these biases and improve decisions. The identification of judgment mistakes is a diagnostic element that will lead to better choices [20].

As an extension of D. Kahneman’s approach, revealing the implicit factors (i.e. the cognitive biases) influencing HTA deliberative processes and discussing about them is a way to mitigate their impact and to improve HTA recommendations. HTA practitioners may not be fully aware of the presence of these factors and their impact on the final recommendations.

Equity and fairness have been repeatedly identified as critical yet often underrepresented considerations in HTA deliberations. Policymakers frequently raise concerns about how to ensure that non-evidence-based factors, such as ethical and societal values, are appropriately recognized without undermining transparency [17]. Studies have shown that while equity is commonly cited as a decision criterion, it often receives less systematic attention than cost-effectiveness and may therefore become marginalized if left implicit [21], [22]. Recent critiques of NICE have also highlighted the risk that insufficient attention to fairness can compromise both procedural legitimacy and allocative justice [23].

In parallel, international efforts such as the HTAi Global Policy Forum have emphasized the importance of inclusive, transparent, and impartial deliberative processes to safeguard fairness in HTA decision-making [24].

By making implicit considerations more explicit, structured tools such as the RELIANT checklist directly support these principles, complementing existing HTA guidelines and providing a practical mechanism to improve objectivity, fairness, legitimacy in the HTA deliberative process. This approach aligns with other frameworks that underscore the importance of uncovering implicit value judgments, such as notions of fairness, through a systematic checklist that shape HTA decisions but often remain unspoken [26].

Under the European regulation on HTA (26), the Joint Clinical Assessment (JCA) is designed to be exempt from value judgments to respect the responsibilities of Member States [26] In this sense, our checklist is a valuable tool that, if systematically used by those responsible for JCA, might contribute to achieve the objective of delivering a European standardized assessment for which value judgements (among other factors) would be identified and hence, controlled.

The authors recommend that HTA practitioners become familiar with the entirety of the checklist prior to the first use. For this, it would be beneficial to share the tool with the the committee members involved in the deliberation and be used in internal trainings as part of continued education within the HTA bodies.

Since the first part of the validation (content validation) was already undertaken and this led us to the actual version of our checklist, a next step would be to perform a pilot test applying the checklist in HTA agencies, followed by a survey to gather feedback following its application in real settings.

## 6 Strenghts and limitations

Our checklist is the first one that accounts for the potential implicit factors influencing the HTA deliberative process.

It has been built upon the findings from two previous research projects and it has been reviewed and endorsed by different experts from five different countries and with different roles in the HTA deliberative process.

The information extracted from the interviews was relevant and comprehensive to the extent that the experts who reviewed and provided feedback on RELIANT considered it an interesting and optimal tool to be used in their countries.

The checklist has the potential to support the HTA deliberative processes at a supranational, national, regional, or local level prior to the HTA deliberation.

While previous work [25] has employed a checklist-based approach to explore fairness in policy, there remains a gap in standardized tools for systematically identifying implicit factors in HTA. On this note, the RELIANT checklist aims to address this gap.

As per the limitations of the checklist, since the tool has been built upon previous research focused on specific countries and in medicines, the applicability in other countries and other health technologies may need to be tested. However, we do not expect much variability since the HTA process is largely common across technologies.

Second, while the checklist cannot capture every undisclosed implicit factor, it is designed to prompt deliberators to critically reflect on a broad range of influencing factors. We also acknowledge that conducting trade-offs between quantified and unquantified effects is a complex and critical aspect of HTA decision-making. However, the primary objective of our work was to highlight the presence and influence of implicit factors and to propose a structured approach to ensure they are recognized and documented, an essential first step before their relative impact can be formally quantified.

Finally, the checklist was tested by a limited number of experts (n = 8) all of whom had previously participated in interviews as part of an earlier project from our research group.[11] Their prior involvement may have introduced bias; however, the validation phase we propose is intended to mitigate this concern.

While the RELIANT checklist represents an initial step toward systematically identifying implicit factors in HTA deliberative processes, further research is needed to refine and validate its use. This includes assessing the relative importance of implicit versus explicit factors in HTA decision-making, identifying which implicit factors truly influence recommendations (to distinguish relevant from irrelevant ones), and exploring in greater depth key elements such as equity and fairness. Such work would help strengthen the robustness and applicability of the RELIANT checklist across diverse HTA contexts.

## CRediT authorship contribution statement

**Clara Monleón:** Writing – review & editing, Writing – original draft, Supervision, Project administration, Methodology, Investigation, Formal analysis, Data curation, Conceptualization. **Hans Martin Späth:** Writing – review & editing, Validation, Supervision, Methodology. **Carlos Crespo:** Supervision, Conceptualization. **Mondher Toumi:** Supervision, Conceptualization.

## Declaration of competing interest

The authors declare that they have no known competing financial interests or personal relationships that could have appeared to influence the work reported in this paper.

## Footnotes

Appendix A Supplementary data to this article can be found online at https://doi.org/10.1016/j.hpopen.2025.100149.

## References

[1] Gauvin FP. What is a Deliberative Process? Institut national de santé publique du Québec [Internet]. 2009 Oct [cited 2022 Aug 29]; Available from: https://www.ncchpp.ca/docs/DeliberativeDoc1_EN_pdf.pdf.

[2] Oortwijn W, Husereau D, Abelson J, Barasa E, Bayani D (Dana), Canuto Santos V, et al. Designing and Implementing Deliberative Processes for Health Technology Assessment: A Good Practices Report of a Joint HTAi/ISPOR Task Force. 2022. Value in Health [Internet]. 2022 Jun;25(6):869–86. Available from: https://linkinghub.elsevier.com/retrieve/pii/S1098301522001607. 10.1016/j.jval.2022.03.018 PMC7613534 35667778

[3] Bond K., Stiffell R., Ollendorf D.A. (2020). Principles for deliberative processes in health technology assessment. Int J Technol Assess Health Care.

[4] Daniels N., Van Der Wilt G.J. (2016). Health technology assessment, deliberative process, and ethically contested issues. Int J Technol Assess Health Care.

[5] EUnetHTA. HTA Core Model® for Work Package 5 Rapid Relative Effectiveness Assessment of EUnetHTA [Internet]. Online. 2013. Available from: http://www.eunethta.eu/sites/5026.fedimbo.belgium.be/files/Model for Rapid REA of pharmaceuticals_final_20130311_reduced.pdf.

[6] Bujar M., McAuslane N., Walker S.R., Salek S. (2019). Quality Decision making in Health Technology Assessment: Issues Facing Companies and Agencies. Ther Innov Regul Sci.

[7] Hofmann B., Cleemput I., Bond K., Krones T., Droste S., Sacchini D. (2014 Mar 30). REVEALING AND ACKNOWLEDGING VALUE JUDGMENTS IN HEALTH TECHNOLOGY ASSESSMENT. Int J Technol Assess Health Care.

[8] Flume M., Bardou M., Capri S., Sola-Morales O., Cunningham D., Levin L.A. (2018 Jan). Approaches to manage ‘affordability’ of high budget impact medicines in key EU countries. J Mark Access Health Policy.

[9] Kristensen F.B., Husereau D., Huić M., Drummond M., Berger M.L., Bond K. (2019 Jan 1). Identifying the need for Good Practices in Health Technology Assessment: Summary of the ISPOR HTA Council Working Group Report on Good Practices in HTA. Value Health.

[10] Rya B., Luce B.R. (2010). EBM, HTA, and CER: Clearing the confusion. Milbank Q.

[11] Monleón C., Martin-Späth H., Crespo C., Dussart C., Toumi M. (2023 Dec). Implicit factors influencing the HTA deliberative processes in 5 European countries: results from a mixed-methods research. Health Policy Open [Internet].

[12] Monleón C., Späth H.M., Crespo C., Dussart C., Toumi M. (2022). Systematic literature review on the implicit factors influencing the HTA deliberative process in Europe. J Mark Access Health Policy.

[13] Monleon Bonet C. HTA DELIBERATIVE PROCESSES IN WESTERN EUROPE : ANALYSIS OF THE INFLUENCE OF IMPLICIT FACTORS AND DEVELOPMENT OF A CHECKLIST TO REVEAL THEM. [Lyon]: Université Lyon 1; 2023.

[14] Baltussen R, Jansen M, Oortwijn W. Evidence-Informed Deliberative Processes for Legitimate Health Benefit Package Design − Part I: Conceptual Framework. Int J Health Policy Manag. 2021 Nov 10. 10.34172/ijhpm.2021.158 PMC9808261 34923808

[15] Baltussen R., Paul Maria Jansen M., Bijlmakers L., Grutters J., Kluytmans A., Reuzel R.P. (2017 Feb 1). Value Assessment Frameworks for HTA Agencies: the Organization of Evidence-Informed Deliberative Processes. Value Health.

[16] Blair I V, Steiner JF, Havranek EP. Unconscious (Implicit) Bias and Health Disparities: Where Do We Go from Here? Background: What We Know So Far [Internet]. Vol. 15, The Permanente Journal/ Spring. 2011. Available from: https://implicit.harvard.edu. 10.7812/tpp/11.979 PMC3140753 21841929

[17] Culyer A.J., Bombard Y. (2012 May). An equity framework for health technology assessments. Med Decis Making.

[18] Oortwijn W, Husereau D, Abelson J, Barasa E, Intervention H, Hock SS, et al. Deliberative Processes for Health Technology Assessment – Report of the HTAi / ISPOR Deliberative Processes for HTA Task Force Methods : The Joint Task Force of Health Technology Assessment International (HTAi). 0–3 p.

[19] Bond K. (2020). Deliberative Processes in Health Technology Assessment: 11 prospects. Problems, and Policy Proposals.

[20] Kahneman D. (2011).

[21] Guindo L.A., Wagner M., Baltussen R., Rindress D., van Til J., Kind P. (2012). From efficacy to equity: Literature review of decision criteria for resource allocation and healthcare decisionmaking. Cost Effectiveness and Resource Allocation.

[22] Shmueli A. (2017). Do the equity-efficiency preferences of the Israeli Basket Committee match those of Israeli health policy makers?. Isr J Health Policy Res.

[23] Charlton V. (2020 Sep 1). NICE and Fair? Health Technology Assessment Policy under the UK’s National Institute for Health and Care Excellence, 1999–2018. Health Care Anal.

[24] Oortwijn W., Jansen M., Baltussen R. (2020 Jan 1). Use of evidence-informed deliberative processes by health technology assessment agencies around the globe. Int J Health Policy Manag.

[25] Culyer AJ. CHE Research Paper 192 Questions of Fairness in Health and Social Care Policy Decisions-A Socratic Approach Questions of Fairness in Health and Social Care Policy Decisions-A Socratic Approach [Internet]. 2023. Available from: www.york.ac.uk/che.

[26] Official Journal of the European Union. (Legislative acts) REGULATIONS REGULATION (EU) 2021/2282 OF THE EUROPEAN PARLIAMENT AND OF THE COUNCIL of 15 December 2021 on health technology assessment and amending Directive 2011/24/EU (Text with EEA relevance).

