# Supplementary material for: The RELIANT checklist: a novel approach to revealing implicit factors in HTA deliberations
Source: Health Policy Open. 2025 Oct 12;10:100149. doi: 10.1016/j.hpopen.2025.100149 (PMC12664409; doi:10.1016/j.hpopen.2025.100149)
Supplement: Supplementary Data 1 [file mmc1.docx]

# Appendix 1 Methods for the Targeted Literature Review and Checklist

# Targeted literature review (TLR)

A TLR was done by developing a search strategy in PubMed. Retrieval was not limited by publication date. The search was performed in September 2022. No geographic limits were applied.

The search strategy was the following one: ("checklist"[Title/Abstract] AND ("HTA"[Title/Abstract] OR "health technology assessment"[Title/Abstract]) AND "deliberat*"[All Fields]) AND (english[Filter] OR french[Filter] OR german[Filter] OR italian[Filter] OR spanish[Filter])

The inclusion criteria comprised:

- Original research publications describing the development or application of a checklist to assist the HTA deliberative process in English, French, German, Italian or Spanish

The exclusion criteria comprised:

- Papers written in languages other than English, French, German, Italian or Spanish
- Narrative reviews not intended to answer research questions about checklists to support the HTA process
- Systematic reviews and meta-analysis
- Papers focused on specific diseases
- Papers focused on health technologies other than pharmaceuticals

The title and abstract of retrieved citations were reviewed by two researchers (CM and MT) to determine their conformity with the inclusion and exclusion criteria. The full text of citations conforming with the inclusion criteria were reviewed independently by them, and when discrepancies were found a third reviewer (CC) performed the assessment to reach a consensus.

# Development of the first version of the checklist

To develop our checklist, we leveraged the findings from a SLR previously published (1), the mixed-methods research described elsewhere (2) and the results of the assessment of the HTAi/ISPOR checklist (3).

Two additional articles (4,5) on implicit factors were proposed proactively by one expert at the time of the interviews. These articles were not retrieved as a result of our SLR because one of them was out of the pre-specified time window of publication (2021) and the other did not match the key words of our search strategy. For the definition of cognitive biases, we leveraged these articles.

The checklist development was based on the “Guidelines for developing evaluation checklists: the checklists development checklist (CDC) by D. L. Stufflebeam” (6).

As a first step, the article research team agreed on the objective of the checklist, defined the checklist’s intended users, studied the relevant literature, and clarified the criteria that should be met by the tool. The objective was to support the HTA practitioners to become aware of the implicit factors that could have an impact on the HTA recommendations. Hence, to improve the deliberation by making it more rationale, impartial, legitimate and transparent.

The checklist had to be easy to understand, easy to implement and reproducible.

We then identified a list of categories that would form the skeleton of the checklist. We focused mainly on the cognitive biases, defined as a systematic deviation from rationality in decision-making or judgment (7) , since these are the implicit factors more subjective prone to biases at the end of the deliberative process and therefore, likely to affect the recommendations.

The selection of these categories was made based on the results from the qualitative research (2) and on what we considered that was missing in the HTAi/ISPOR checklist (3) from an implicit factors standpoint.

In a third step, we defined the order of these categories going from a more general and objective approach to finalize with the cognitive biases. For each category, we developed a set of questions with the intention to help the HTA practitioner to reflect on the factors that they considered in the deliberative process.

We also defined the format of answers with options of “yes/no/sometimes” and “low/medium/high”. We believe this kind of format contributes to the understanding of the checklist and simplifies its use.

**Assessment of the checklist by the experts**

Finally, we agreed on a first version ready to be shared with a group of experts that was appointed for the content validity phase. The interviewees were part of the group who participated in the previous mixed-methods research. The group was selected based on the acceptance in participating in the development of the checklist. The final group was composed by eight experts representing the five European countries (France, Germany, Italy, Spain, United Kingdom) and of different profiles of HTA Decision-makers (Table 1a). There was at least one expert per country and per type of profile.

Two rounds of review were performed (November 2022 and January 2023). The first round aimed to get input into the objectives of the checklist, target audience, time of review, and categories with their respective questions. The second and final version was shared to make sure the experts felt comfortable with it after the changes were integrated. Experts were asked individually via e-mail to provide feedback on the different sections of the first version of the checklist, the items, the feasibility of its implementation as well as the place of the checklist in the deliberative process.

An open question was asked about their overall perception on the checklist. A follow-up teleconference was proposed to clarify any question. Two out of the eight experts (one from France and one from the UK) accepted this option.

As a result of the review, all comments were incorporated and a second and final version was consolidated.

The experts were asked where they would see the implementation of the checklist in the HTA deliberative process.

Table 1a. Profile characteristics of the eight study participants

| **Country** | **Organization from which experience was gained** |
| --- | --- |
| France | HAS^1^ |
| France | HAS |
| Germany | PKV^2^ |
| Italy | AGENAS^3^ |
| Spain | Malvarrosa Hospital Clinical Department |
| UK | NICE^4^ |
| UK | NICE |
| UK | NHS^5^ |

^1^French National Authority (“Haute Autorité de Santé”), ^2^Joint Federal Association of Private Health Insurance (“Verband der Privaten Krankenversicherung) ^3^ National Agency for the Regional healthcare services, HTA department (“Agenzia Nazionale per i Servici Sanitari Regionali”), ^4^United Kingdom National Health Service, ^5^National Institute for Health and Care Excellence

**Appendix 2** Assessment of the different phases of the HTAi/ISPOR checklist

| **Phase** | **Strengths** | **Limitations** |
| --- | --- | --- |
| Determining the need for a deliberative process | The scope of the deliberation provides the overall context and proposes two different settings (deliberation about the process and within the process) and outlines the characteristics of each of them. It sets the needs for a deliberative process: reasons to deliberate, desired outcomes and scope of the deliberation. In the reasons to deliberate it considers an implicit factor (“self-interest”). | The ultimate goal of “facilitating participatory decision making” was not included in the reasons to deliberate. |
| Preparing for a deliberative process | This section describes the contextual factors and guidance to be considered for the implementation. This enables planning and structurisation. It also helps HTA practitioners to have consciousness on what can hamper or drive the process. | It is unclear to what extent this section helps the HTA practitioners to prepare for the deliberative process. It covers one type of implicit factor (contextual factors). There may be additional implicit factors other than contextual factors (e.g., biases) that play a role in the implementation and that were not considered in the checklist. |
| Conducting a deliberative process | - It reinforces the diversity and the broad spectrum of stakeholders’ perspectives. - It reveals the importance of determining the type of membership. - It reveals the importance of transparency (openness of deliberation and type of information shared with the public) - It highlights the importance of defining a way to reach the end of the deliberation (the recommendation) - It remarks the importance of following and placing in the public domain the explicit criteria. | - The perspective on memberships, and the selection of profile characteristics should be part of the previous section (” preparing for a deliberative process”) since they should be well defined before conducting the deliberative process. - The authors focus on the contextual factors relevant to the process (internal and external) and on the explicit criteria. There is no dedicated attention to other implicit factors (e.g., ethics, cognitive biases), which is a major limitation of the checklist. |
| Supporting a deliberative process | - It focuses on the type of information and ways to make it available to the HTA participants. A clear understanding of the information is critical to ensure meaningful participation. | - Regarding the type of information that will be made available to the deliberative process, it is rather general (e.g., “all information made available to the HTA body”). - It is not clear how the information will be reviewed and why the distinction between HTA body and participants was made, as the HTA bodies are the target audience. One of the answers to the question “what information will be considered by participants?” was “any information provided by HTA body and participants”. There are no questions to check if all relevant information is available to the participants or if the information was clearly understood. |
| Development and communication of the outputs of deliberation | - Developing a communication strategy and defining what and how will be shared with the public is key to ensure the transparency of the process. This will enable the understanding of all the elements that were considered for the recommendation. - It focuses on the way to communicate the outputs. | There is no mention to the timelines associated to the communication of the outputs. |
| Monitoring and evaluation. | This section is important to check if the deliberative process is achieving its intended goals. The table shows a list of process and outcomes indicators along with proposed methods that can help the HTA body to assess and evaluate the deliberative process. | The table proposing different indicators and methods is not integrated in the checklist and it is not clear how this information as a part of the checklist is going to be available to those interested. |

**Appendix 3 The RELIANT checklist version prior to the experts’ review**

| Sections | Questions | | |
| --- | --- | --- | --- |
| 1. Perspectives | - What are the perspectives that have been considered?   *(select one or more):*  Health insurer  Societal  Other __________________ | | |
| 1. Objective factors   (explicit or implicit in  the HTA framework) | *What is the importance that you allocate to these factors?* | | *How much influence did they have in your recommendation?* |
|  | - Clinical burden (e.g., severity of the disease, mortality, frequency of hospitalizations) | \| Low \| Medium \| High \| \| --- \| --- \| --- \| \|  \|  \|  \| | \| Low \| Medium \| \| High \| \| --- \| --- \| --- \| --- \| \|  \|  \|  \| \| |
|  | - Humanistic   burden (impact on quality of life of patients’ and caregivers) | \| Low \| Medium \| High \| \| --- \| --- \| --- \| \|  \|  \|  \| | \| Low \| Medium \| High \| \| --- \| --- \| --- \| \|  \|  \|  \| |
|  | - Economic burden:   direct costs of treatment | \| Low \| Medium \| High \| \| --- \| --- \| --- \| \|  \|  \|  \| | \| Low \| Medium \| High \| \| --- \| --- \| --- \| \|  \|  \|  \| |
|  | - Economic burden: indirect costs of   treatment | \| Low \| Medium \| High \| \| --- \| --- \| --- \| \|  \|  \|  \| | \| Low \| Medium \| High \| \| --- \| --- \| --- \| \|  \|  \|  \| |
|  | - Prevalence and incidence (rare disease, disease affecting large number of   patients) | \| Low \| Medium \| High \| \| --- \| --- \| --- \| \|  \|  \|  \| | \| Low \| Medium \| High \| \| --- \| --- \| --- \| \|  \|  \|  \| |
|  | - Vulnerability of   the population | \| Low \| Medium \| High \| \| --- \| --- \| --- \| \|  \|  \|  \| | \| Low \| Medium \| \| High \| \| --- \| --- \| --- \| --- \| \|  \|  \|  \| \| |
|  | - Availability of effective alternatives (on and off-label)/unmet need | \| Low \| Medium \| High \| \| --- \| --- \| --- \| \|  \|  \|  \| | \| Low \| Medium \| High \| \| --- \| --- \| --- \| \|  \|  \|  \| |
|  | - Innovativeness of the technology independent of the performance | \| Low \| Medium \| High \| \| --- \| --- \| --- \| \|  \|  \|  \| | \| Low \| Medium \| High \| \| --- \| --- \| --- \| \|  \|  \|  \| |
|  | - Strength of the evidence (robustness of clinical study design and effect size) | \| Low \| Medium \| High \| \| --- \| --- \| --- \| \|  \|  \|  \| | \| Low \| Medium \| High \| \| --- \| --- \| --- \| \|  \|  \|  \| |
|  | - Clinical sign of potential value, but value not fully demonstrated with evidence (aspirational   benefit demonstrated by immature data, surrogate,  partially validated endpoints, single arm study)*  **To be rated only if*  *the strength of the evidence was considered as medium or low* | \| Low \| Medium \| High \| \| --- \| --- \| --- \| \|  \|  \|  \| | \| Low \| Medium \| High \| \| --- \| --- \| --- \| \|  \|  \|  \| |
|  | - Strong biological hypothesis of efficacy (hypothesis well supported by preclinical research, physiology, pharmacology, disease mechanism) *   **To be rated only if*  *the strength of the evidence was considered as medium or low* | \| Low \| Medium \| High \| \| --- \| --- \| --- \| \|  \|  \|  \| | \| Low \| Medium \| High \| \| --- \| --- \| --- \| \|  \|  \|  \| |
| 1. Meeting dynamics | *Did you receive all information necessary to make an*  *informed decision?* | | \| Yes \| No \| Sometimes \| \| --- \| --- \| --- \| \|  \|  \|  \| |
|  | *Could the way the data was presented have affected your perception?* | | \| Yes \| No \| Sometimes \| \| --- \| --- \| --- \| \|  \|  \|  \| |
|  | *Did you ask all relevant question from your perspective?* | | \| Yes \| No \| Sometimes \| \| --- \| --- \| --- \| \|  \|  \|  \| |
|  | *Did you receive clear and unambiguous responses to your questions?* | | \| Yes \| No \| Sometimes \| \| --- \| --- \| --- \| \|  \|  \|  \| |
|  | *Did any dominant perspective/opinion prevent alternative view to discussion?* | | \| Yes \| No \| Sometimes \| \| --- \| --- \| --- \| \|  \|  \|  \| |
|  | *Could the way the decision was made (for example consensus vs. voting procedure, show if hands) have affected your decision?* | | \| Yes \| No \| Sometimes \| \| --- \| --- \| --- \| \|  \|  \|  \| |
| 1. Personal interests | *Do you believe that your personal interests (e.g., willingness to use the drug by yourself/your friends/your relatives/your patients) could have any impact on your perception?* | | \| Yes \| No \| Sometimes \| \| --- \| --- \| --- \| \|  \|  \|  \| |
| 1. Previous experiences | *Was your perception impacted by your previous experience*  *with the manufacturer?* | | \| Yes \| No \| Sometimes \| \| --- \| --- \| --- \| \|  \|  \|  \| |
|  | *Was your perception impacted by your previous experience*  *with the disease?* | | \| Yes \| No \| Sometimes \| \| --- \| --- \| --- \| \|  \|  \|  \| |
|  | *Was your perception impacted by your previous experience*  *with similar type of medication?* | | \| Yes \| No \| Sometimes \| \| --- \| --- \| --- \| \|  \|  \|  \| |
| 1. Context | *Do you believe that the final decision was impacted by any*  *form of pressure listed below?* | | *What impact did it have on the final decision?* |
|  | - Political | \| Yes \| No \| Sometimes \| \| --- \| --- \| --- \| \|  \|  \|  \| | \| Low \| Medium \| High \| \| --- \| --- \| --- \| \|  \|  \|  \| |
|  | - Patient groups   and caregivers | \| Yes \| No \| Sometimes \| \| --- \| --- \| --- \| \|  \|  \|  \| | \| Low \| Medium \| High \| \| --- \| --- \| --- \| \|  \|  \|  \| |
|  | - Healthcare professionals | \| Yes \| No \| Sometimes \| \| --- \| --- \| --- \| \|  \|  \|  \| | \| Low \| Medium \| High \| \| --- \| --- \| --- \| \|  \|  \|  \| |
|  | - Pharmaceutical companies | \| Yes \| No \| Sometimes \| \| --- \| --- \| --- \| \|  \|  \|  \| | \| Low \| Medium \| High \| \| --- \| --- \| --- \| \|  \|  \|  \| |
|  | - Social | \| Yes \| No \| Sometimes \| \| --- \| --- \| --- \| \|  \|  \|  \| | \| Low \| Medium \| High \| \| --- \| --- \| --- \| \|  \|  \|  \| |
|  | - Cultural | \| Yes \| No \| Sometimes \| \| --- \| --- \| --- \| \|  \|  \|  \| | \| Low \| Medium \| High \| \| --- \| --- \| --- \| \|  \|  \|  \| |
|  | - Media | \| Yes \| No \| Sometimes \| \| --- \| --- \| --- \| \|  \|  \|  \| | \| Low \| Medium \| \| High \| \| --- \| --- \| --- \| --- \| \|  \|  \|  \| \| |
|  | - Administration/   Health authorities | \| Yes \| No \| Sometimes \| \| --- \| --- \| --- \| \|  \|  \|  \| | \| Low \| Medium \| \| High \| \| --- \| --- \| --- \| --- \| \|  \|  \|  \| \| |
|  | - Moral/ethic   (your sense of  equity and values) | \| Yes \| No \| Sometimes \| \| --- \| --- \| --- \| \|  \|  \|  \| | \| Low \| Medium \| High \| \| --- \| --- \| --- \| \|  \|  \|  \| |
|  | - Healthcare   system affordability | \| Yes \| No \| Sometimes \| \| --- \| --- \| --- \| \|  \|  \|  \| | \| Yes \| No \| Sometimes \| \| --- \| --- \| --- \| \|  \|  \|  \| |
|  | - Allocated budget to certain diseases | \| Yes \| No \| Sometimes \| \| --- \| --- \| --- \| \|  \|  \|  \| | \| Yes \| No \| Sometimes \| \| --- \| --- \| --- \| \| ☐ \|  \|  \| |
|  | - Feasibility (ability   of the healthcare  system to ensure a right use of treatment) | \| Yes \| No \| Sometimes \| \| --- \| --- \| --- \| \|  \|  \|  \| | \| Yes \| No \| Sometimes \| \| --- \| --- \| --- \| \|  \|  \|  \| |
|  | - Public health   priority | \| Yes \| No \| Sometimes \| \| --- \| --- \| --- \| \|  \|  \|  \| | \| Yes \| No \| Sometimes \| \| --- \| --- \| --- \| \|  \|  \|  \| |
|  | Other__________ | | \| Yes \| No \| Sometimes \| \| --- \| --- \| --- \| \|  \|  \|  \| |
| 1. Cognitive   biases and  fallacies (individual  and collective) | *In an effort to be as objective as possible, do you consider any*  *of these potential biases may have impacted your judgement?* | | \| Yes \| No \| Sometimes \| \| --- \| --- \| --- \| \|  \|  \|  \| |
|  | - Affect heuristic (decision influenced by subjective feelings) | | \| Yes \| No \| Sometimes \| \| --- \| --- \| --- \| \|  \|  \|  \| |
|  | - Anchoring bias (focusing on the first picture you learn and staying with the first impression) | | \| Yes \| No \| Sometimes \| \| --- \| --- \| --- \| \|  \|  \|  \| |
|  | - Attentional bias (paying disproportional attention to some things, over others) | | \| Yes \| No \| Sometimes \| \| --- \| --- \| --- \| \|  \|  \|  \| |
|  | - Authority bias (having more confidence in a decision that was validated by an authority figure) | | \| Yes \| No \| Sometimes \| \| --- \| --- \| --- \| \|  \|  \|  \| |
|  | - Automation bias (tendency to accept automated clinical decision advice) | | \| Yes \| No \| Sometimes \| \| --- \| --- \| --- \| \|  \|  \|  \| |
|  | - Availability bias (making judgments of likelihood based on ease of recall) | | \| Yes \| No \| Sometimes \| \| --- \| --- \| --- \| \|  \|  \|  \| |
|  | - Confirmation bias (focusing on information confirming personal beliefs) | | \| Yes \| No \| Sometimes \| \| --- \| --- \| --- \| \|  \|  \|  \| |
|  | - Consistency tendency (difficulty in changing a point of view when the change is justified by the new evidence) | | \| Yes \| No \| Sometimes \| \| --- \| --- \| --- \| \|  \|  \|  \| |
|  | - False consensus effect (overestimation on how much other people are aligned with your appreciation) | | \| Yes \| No \| Sometimes \| \| --- \| --- \| --- \| \|  \|  \|  \| |
|  | - Framing effects (perception influenced by the way in which the evidence was presented) | | \| Yes \| No \| Sometimes \| \| --- \| --- \| --- \| \|  \|  \|  \| |
|  | - Groupthink (situation in which the views of the decision-making group become homogeneous and contrary views are discouraged) | | \| Yes \| No \| Sometimes \| \| --- \| --- \| --- \| \|  \|  \|  \| |
|  | - Halo effect (giving disproportionate weight in decision making to the beliefs of eminent individuals) | | \| Yes \| No \| Sometimes \| \| --- \| --- \| --- \| \|  \|  \|  \| |
|  | - Hard/bandwagon effect (choosing based on other people choices) | | \| Yes \| No \| Sometimes \| \| --- \| --- \| --- \| \|  \|  \|  \| |
|  | - In-group conformity (having more confidence in a decision when it is in agreement with others) | | \| Yes \| No \| Sometimes \| \| --- \| --- \| --- \| \|  \|  \|  \| |
|  | - Intellectual bias (becoming closed-minded about a belief) | | \| Yes \| No \| Sometimes \| \| --- \| --- \| --- \| \|  \|  \|  \| |
|  | - Novelty (uncritical acceptance of a new technology) | | \| Yes \| No \| Sometimes \| \| --- \| --- \| --- \| \|  \|  \|  \| |
|  | - Optimism bias (tendency to overestimate the likelihood of favourable outcomes) | | \| Yes \| No \| Sometimes \| \| --- \| --- \| --- \| \|  \|  \|  \| |
|  | - Overconfidence bias (overestimation of personal ability or opinion) | | \| Yes \| No \| Sometimes \| \| --- \| --- \| --- \| \|  \|  \|  \| |
|  | - Reductionism bias (reducing complex scenarios into simpler ideas) | | \| Yes \| No \| Sometimes \| \| --- \| --- \| --- \| \|  \|  \|  \| |
|  | - Scientific inbreeding (replication of views by the individuals with the same training/educational experience) | | \| Yes \| No \| Sometimes \| \| --- \| --- \| --- \| \|  \|  \|  \| |
|  | - Search satisficing (stopping research once self-satisfying evidence is found) | | \| Yes \| No \| Sometimes \| \| --- \| --- \| --- \| \|  \|  \|  \| |
|  | - Specialty bias (form of groupthink, situation when those in a specialty adopt a homogeneous set of beliefs) | | \| Yes \| No \| Sometimes \| \| --- \| --- \| --- \| \|  \|  \|  \| |
|  | - Thinking inside the box (inflexibility of thinking within decision-making group) | | \| Yes \| No \| Sometimes \| \| --- \| --- \| --- \| \|  \|  \|  \| |
|  | - Fallacy of silence (omission of contradictory evidence) | | \| Yes \| No \| Sometimes \| \| --- \| --- \| --- \| \|  \|  \|  \| |
|  | - Planning fallacy (incorrect estimation of the benefits and timing of policies/actions) | | \| Yes \| No \| Sometimes \| \| --- \| --- \| --- \| \|  \|  \|  \| |
|  | - Sunk-cost fallacy (making decision based on previously spent effort for the assessment, unwillingness to change the course when too much effort has been invested) | | \| Yes \| No \| Sometimes \| \| --- \| --- \| --- \| \|  \|  \|  \| |
|  | - Arguing from ignorance (claiming that a statement is false because we do not know if it is true and vice versa) | | \| Yes \| No \| Sometimes \| \| --- \| --- \| --- \| \|  \|  \|  \| |
|  | - Fallacy of diversion (substitution of one argument for another) | | \| Yes \| No \| Sometimes \| \| --- \| --- \| --- \| \|  \|  \|  \| |
|  | - Fallacy of division (reasoning applied to a group is confused with reasoning applied to individual) | | \| Yes \| No \| Sometimes \| \| --- \| --- \| --- \| \|  \|  \|  \| |
|  | - Appealing to authority (giving inappropriate weight to the argument coming from a source of expertise or authority | | \| Yes \| No \| Sometimes \| \| --- \| --- \| --- \| \|  \|  \|  \| |
|  | - Appealing to popularity (claiming that the argument is valid base on most people’s acceptance) | | \| Yes \| No \| Sometimes \| \| --- \| --- \| --- \| \|  \|  \|  \| |
|  | - Ad hominem fallacy (directing criticism to a person rather than criticize the argument itself) | | \| Yes \| No \| Sometimes \| \| --- \| --- \| --- \| \|  \|  \|  \| |
|  | - Straw man fallacy (attacking a misrepresentation of an opinion) | | \| Yes \| No \| Sometimes \| \| --- \| --- \| --- \| \|  \|  \|  \| |

**Appendix 4 Feedback on the RELIANT checklist from the eight HTA experts**

| **Experts’ countries** | **Main comments on the content** | **Changes reflected in the updated version of the checklist** | **Overall perceptions on the checklist** | **Position of the checklist in the HTA process** |
| --- | --- | --- | --- | --- |
| Italy | “I am afraid of its length since this could difficult is implementation.”  “I don’t see so much relevance to the part dedicated to fallacies.” | The checklist was shortened following the removal of the part focused on “fallacies” | “This tool is very interesting given its exhaustiveness.”  “I put a face to some of the referred biases.” | Pre-appraisal |
| France | “The part related to fallacies could be removed to shorten the checklist and make it more practical.” | The checklist was shortened following the removal of the part focused “fallacies.” | “This is a very complete checklist with many considerations on it. It is exhaustive but practical at the same time.”  “The checklist could be used in some workshops first to make the committee aware of its use.” | Pre-appraisal |
| France | “It would be helpful to make it shorter to facilitate the implementation”. | The checklist was shortened following the removal of the part focused “fallacies” | “This checklist is comprehensive and innovative.”  “I like the fact that both group and individual dynamics are being considered.” | Pre-appraisal |
| Germany | “From a German system’s perspective, it would be implemented in the assessment but not in coverage and pricing discussions.”  “Maybe in Section C, add “committee “to make it clearer”. | The name of Section C was further detailed. It was added “committee” to “meeting dynamics”. | “The list is fairly comprehensive.” | During the assessment |
| Spain | “It would be good to specify the context: what are the technologies (drugs, medical devices, vaccines?) subject for the checklist.” | It was clarified that the checklist it is intended to be applied only to medicines. | “I found the checklist interesting, and I would be eager to see how this is intended to be implemented.” | Pre-appraisal |
| UK | “At end maybe add an open space for any more comment – qualitative evidence/ information can often be very useful.”  “Is the checklist meant to be completed prospectively or retrospectively?” | A row was added to integrate any additional comment that the expert may have.  The intend of use of the checklist was defined (prospectively). | “Very nice tool be self-aware of the biases and other factors present in the deliberative process.” | Post appraisal |
| UK | “Maybe clarify in introductory notes about the checklist intention for the specific person/ persons completing it.”  “Add a row for strength of cost-effectiveness evidence.”  “SMC and some other HTA’s may give a different rating to patients and caregivers QoL – consider distinguishing between the two options. | The intended users were defined in the description of the checklist.  A row for strength of cost-effectiveness evidence was added in section B).  In Section B, the impact in quality of life was distinguished between patients and caregivers | “Nice checklist. It would be good to pilot the checklist with a few intended users of course.”  “I find section G (Cognitive bias) a very interesting list “ | Pre-appraisal |
| UK | “Clarify the perspectives of whom (section A of the checklist)  “Maybe label Health insurer/payer”  “If the checklist is intended to be used prospectively, use the present tense in the questions instead of past tenses.”  “Maybe it needs clarity on what indirect costs means, e.g., everything else than direct cost of treatment e.g. hospital and other resource use costs, caregiver costs, productivity costs, costs on other government sectors? | It was added into the first question of the checklist (section A): “What are the perspectives that have been **considered by the HTA expert** completing the checklist?”  In the options of answer in Section A, it was added “payer” along to health insurer.  The questions were formulated in present tense.  The clarification on what indirect costs mean was added accordingly in the part of economic burden (Section B) | “Very interesting and helpful checklist. If applied in a systematic way, this could make a big difference in the way the HTA is conducted. It would promote a more objective and conscious exercise.” | Pre-appraisal |
